# Phosphorylation of Histone H2AX in the Mouse Brain from Development to Senescence

**DOI:** 10.3390/ijms15011554

**Published:** 2014-01-21

**Authors:** Serena Barral, Riccardo Beltramo, Chiara Salio, Patrizia Aimar, Laura Lossi, Adalberto Merighi

**Affiliations:** 1 Department of Veterinary Sciences, Università di Torino, Via Leonardo da Vinci 44, Grugliasco I-10095, Italy; E-Mails: chiara.salio@unito.it (C.S.); patrizia.aimar@unito.it (P.A.); 2 Istituto Italiano di Neuroscienze (INN), Via Leonardo da Vinci 44, Grugliasco I-10095, Italy

**Keywords:** H2AX, histone, DNA damage, immunocytochemistry, cell proliferation, apoptosis, neuroprotection, Alzheimer’s disease, Huntington’s disease

## Abstract

Phosphorylation of the histone H2AX (γH2AX form) is an early response to DNA damage and a marker of aging and disease in several cells and tissues outside the nervous system. Little is known about *in vivo* phosphorylation of H2AX in neurons, although it was suggested that γH2AX is an early marker of neuronal endangerment thus opening the possibility to target it as a neuroprotective strategy. After experimental labeling of DNA-synthesizing cells with 5-bromo-2-deoxyuridine (BrdU), we studied the brain occurrence of γH2AX in developing, postnatal, adult and senescent (2 years) mice by light and electron microscopic immunocytochemistry and Western blotting. Focal and/or diffuse γH2AX immunostaining appears in interkinetic nuclei, mitotic chromosomes, and apoptotic nuclei. Immunoreactivity is mainly associated with neurogenetic areas, *i.e.*, the subventricular zone (SVZ) of telencephalon, the cerebellar cortex, and, albeit to a much lesser extent, the subgranular zone of the hippocampal dentate gyrus. In addition, γH2AX is highly expressed in the adult and senescent cerebral cortex, particularly the piriform cortex. Double labeling experiments demonstrate that γH2AX in neurogenetic brain areas is temporally and functionally related to proliferation and apoptosis of neuronal precursors, *i.e.*, the type C transit amplifying cells (SVZ) and the granule cell precursors (cerebellum). Conversely, γH2AX-immunoreactive cortical neurons incorporating the S phase-label BrdU do not express the proliferation marker phosphorylated histone H3, indicating that these postmitotic cells undergo a significant DNA damage response. Our study paves the way for a better comprehension of the role of H2AX phosphorylation in the normal brain, and offers additional data to design novel strategies for the protection of neuronal precursors and mature neurons in central nervous system (CNS) degenerative diseases.

## 1. Introduction

First identified as an isoform of the core histone H2A, H2AX constitutes a major H2A species [1]. The role of H2AX γ-phosphorylation in DNA damage was first suggested by showing that phosphorylated H2AX (γH2AX) appeared rapidly following cell exposure to ionizing radiations [1]. The amount of γH2AX was estimated to be directly related to the number of double strand breaks (DSBs) and, thus, to the extent of DNA damage [2]. In parallel, the development of specific anti-γH2AX antibodies confirmed that H2AX was massively phosphorylated in nuclear foci of chromatin surrounding the DSBs [2]. Because of the rapid induction and amplification of γH2AX, the existence of a dose-related linear increase of foci following exposure to irradiation [3], and the 1:1 correspondence between the number of γH2AX foci and the number of DSBs [4], γH2AX recognizing antibodies became the “gold standard” to histologically detect DSBs. Since these earlier observations, it became clear that γH2AX not only was related to the DNA damage that follows irradiation, but was also associated with senescence and disease in several cells, tissues and organs. In a seminal paper [5], senescing human cells of various origins (normal and WI38 fibroblasts, PrEC: prostate epithelial cells) and five mouse organs (liver, testis, kidney, lung and brain) were shown to display increased levels of H2AX phosphorylation. These observations were subsequently, confirmed and/or extended [6–9], and, in parallel, γH2AX was described in various types of tumors entering the scene of clinical research and therapy in oncology [10,11].

Whereas phosphorylation of H2AX was extensively studied in connection with experimental DNA damage, senescence, and oncogenesis, comparatively little attention has been paid to γH2AX in normal cells, although it was originally reported that γH2AX levels change throughout cell cycle progression in proliferating HeLa cells with an intact DNA [12]. Later, it was concluded that phosphorylation of H2AX assumed the form of a γH2AX focus only in response to DNA DSBs [3]. However, there were several cases of γH2AX formation not due to DSBs, although in these cases γH2AX did not assume a focal pattern [10]. Moreover, while at the beginning it was foci disappearance which was associated with the repair of DNA damage, γH2AX is now also used as a marker for exploring the spatial distribution and the cellular DNA repair kinetics [3,13,14]. Therefore, phosphorylation of H2AX not only is nowadays linked to a DNA damage response (DDR), but also to other yet uncharacterized functions, primarily during cell division and/or apoptosis.

Morphogenesis and cellular differentiation in central nervous system (CNS) take place very early in development. Although there are some remarkable exceptions including the possibility of a replacement in adult life [15], the vast majority of mature neurons is postmitotic, and lacks the capacity of proliferation. As a consequence, neurons in any species, at any given time, are virtually as old as the animal itself. Neurons are among the most metabolically active cells, their gene expression levels being two to three fold higher compared with other cell types [16]. This renders the brain one of the most vulnerable organs in terms of the damage to cellular DNA. Consequently, DDR and repair systems should have great importance not only during development, but also in the adult and old CNS.

Coincident with DNA repair is the activation of a DNA damage-induced signaling pathway that halts the cell cycle while repair occurs, or, alternatively, leads to apoptosis when damage is so extensive as to be incompatible with recovery [17]. Insults not necessarily resulting in neuronal death can induce γH2AX, and a role for H2AX alterations in determining neuronal vulnerability following damage was hypothesized. Not only did these observations suggest a different sensitivity to irradiation between neuronal precursors and mature neurons [18], but γH2AX was also proposed as an early marker of neuronal endangerment after ionotropic glutamate receptor activation and seizures in the adult brain [19,20]. In addition, DDR and H2AX phosphorylation have been implicated, albeit only tentatively, in Alzheimer’s [21] and Huntington’s [22] diseases. Altogether, these findings suggest that novel neuroprotective strategies could stem from specific targeting of the DDR/γH2AX pathway.

We set up this study to map the occurrence of γH2AX in normal mouse brain. We asked whether or not there was a histological and structural relationship between H2AX phosphorylation and the known sites of proliferation/apoptosis during the course of neural development and aging. We were convinced that an answer to the above question could be of help in setting a baseline for further exploitation of neuroprotective strategies tagging H2AX.

## 2. Results and Discussion

### 2.1. Expression of γH2AX in the Mouse Brain

**Figure 1.**
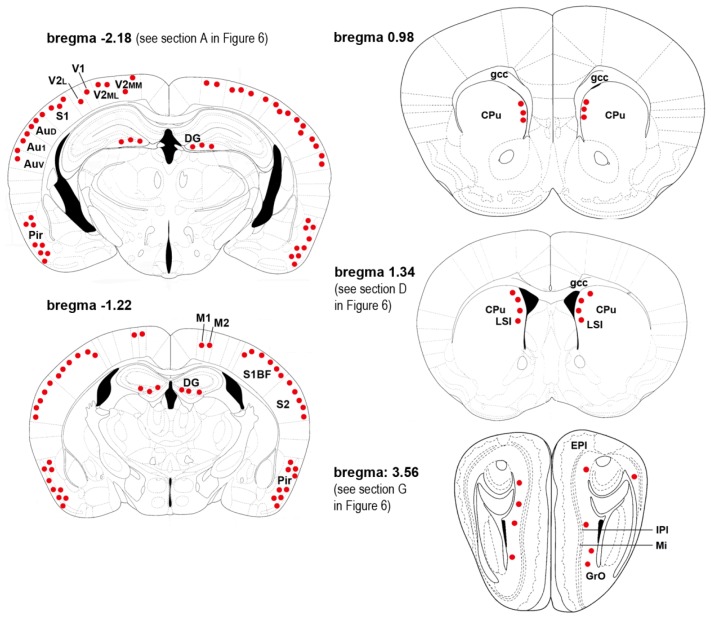
Maps of distribution of γH2AX immunoreactivity (IR) in forebrain. Coronal sections of the mouse brain (adapted from [23]). Red dots indicate the localization of immunoreactive nuclei. Abbreviations: Au1 = primary auditory cortex; AuD = second auditory cortex, dorsal; AuV = second auditory cortex, ventral; CPu = caudate nucleus/putamen; DG = dentate gyrus of hippocampus; EPl = external plexiform layer of the olfactory bulb; gcc = genu, corpus callosum; GrO = granule cell layer of the olfactory bulb; IPl = internal plexiform layer of the olfactory bulb; LSI = intermediate part of the lateral septal nucleus; M1 = primary motor cortex; M2 = second motor cortex; Pir = piriform cortex; Mi = mitral cell layer of the olfactory bulb; S1 = primary somatosensory cortex; S1BF = primary somatosensory cortex, barrel field; S2 = second somatosensory cortex; V1 = primary visual cortex; V2L = secondary visual cortex, lateral area; V2ML = secondary visual cortex, mediolateral area; and V2MM = secondary visual cortex, mediomedial area.

A map of the occurrence and distribution of γH2AX was obtained in developing, postnatal, adult, and senescent mice along the entire rostro-caudal axis of the brain by coronal serial sectioning and immunocytochemistry (ICC) at light microscopy level using two different antibodies raised against H2AX phosphorylated at Ser139. Results of semi-quantitative analysis of distributional data are summarized in Figure 1 and Table 1.

**Figure 2.**
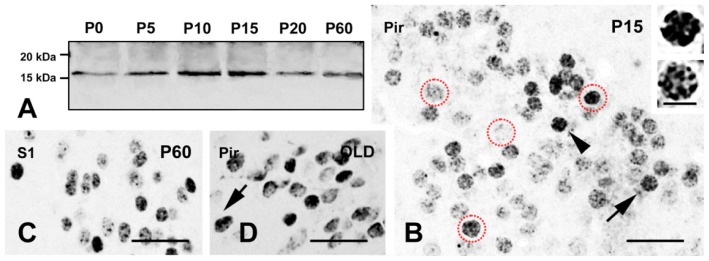

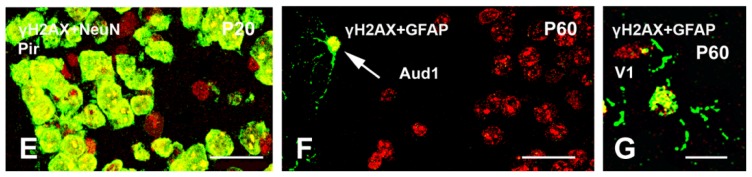
Distribution of γH2AX mouse brain. (**A**) Western blot detection of γH2AX-IR in immunoprecipitates of mouse whole brain extracts; (**B**–**D**) γH2AX IR in the cerebral cortex. Immunostaining displays a nuclear distribution in foci of intense IR (arrows—lower insert in (**B**)) or staining in metaphase chromosomes (arrowhead—upper insert in (**B**)). Four nuclei in (**B**) are encircled by red dots to respectively show, by way of examples, unstained, weakly, moderately, and strongly stained cells; (**E**) Double immunocytochemical labeling for γH2AX (red) and the neuronal marker NeuN (green). most nuclei in piriform cortex are double-labeled (yellow); and (**F**,**G**) Double immunocytochemical labeling for γH2AX (red) and GFAP (green): double-labeled astrocytes (yellow) in the auditory (the arrow in (**F**)) and primary visual cortex (**G**) are apparent together with γH2AX (red) singularly labeled neuronal nuclei. Abbreviations: γH2AX = phosphorylated form of histone H2AX; Aud1 = primary auditory cortex; GFAP = glial fibrillary acidic protein; NeuN = Neuronal nuclei antigen; P = postnatal day; Pir = piriform cortex; S1 = primary somatosensory cortex; and V1 = primary visual cortex. Scale bars: 50 μm; inserts: 10 μm.

The specificity of immunostaining was confirmed with current ICC controls and by detection of γH2AX in whole brain homogenates, after immunoprecipitation and Western blot analysis (Figure 2A).

#### 2.1.1. Forebrain

**Figure 3.**
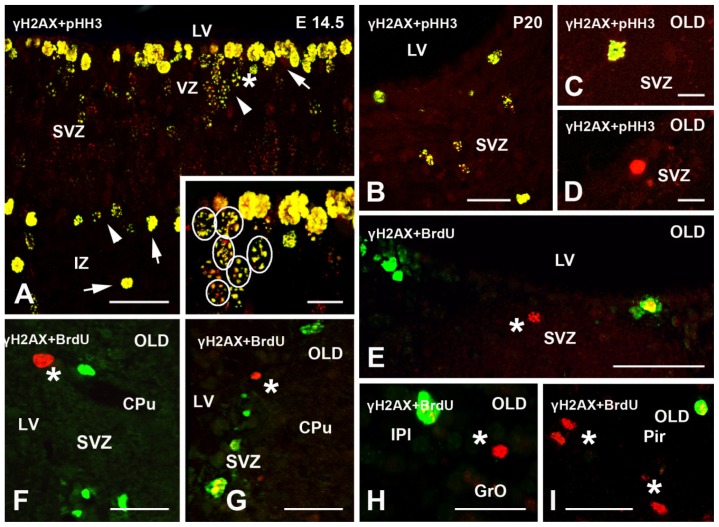
Double immunofluorescence (IMF) labeling of γH2AX, proliferation, and DNA synthesis markers in mouse brain. (**A**–**D**) γH2AX (red) + pHH3 (green) double staining in the SVZ at different ages (reference point A, see Figure 6). At E 14.5 the two populations of proliferating cells in the developing cerebral wall are double-labeled cells (yellow) with M (arrows) and G_2_ phase (arrowheads) morphologies. The area indicated by the asterisk in (**A**) is shown at higher magnification in the insert where the profiles of some G_2_ nuclei are outlined by ovals. Note the focal pattern of staining in G_2_ cells and the very intense labeling of condensed chromosomes in M cells. At P20 double labeled cells are for the most in G_2_. In the SVZ of senescent mice, pHH3 labeled cells are only occasionally seen. A double stained cell in M phase is shown in (**C**), whereas the γH2AX intensely stained cell in (**D**) displays a very condensed nucleus typical of late apoptosis. (**E**–**I**) γH2AX (red) + BrdU (green) double IMF in senescent mice. The γH2AX-IR nuclei (red) are indicated by asterisks and display low-to-mild focal staining or, more frequently, intense staining with chromatin condensation. BrdU-IR cells (green) and double labeled cells (yellow) are detected just below the border of the LV (**E**) SVZ—bregma 0.98; (**F**,**G**) RMS—reference point D; and (**H**) OB (reference point G, see Figure 6) are scattered in cerebral cortex ((**I**) reference point A, see Figure 6). Abbreviations: γH2AX = phosphorylated form of histone H2AX; BrdU = 5-bromo-2-deoxyuridine; CPu = caudate nucleus/putamen; E = embryonic day; GrO = granule cell layer of the olfactory bulb; IPl = internal plexiform layer of the olfactory bulb; IZ = intermediate zone of the cerebral wall; LV = lateral ventricle; pHH3 = phosphorylated form of histone H3; Pir = piriform cortex; SVZ = subventricular zone of the cerebral wall; and VZ = ventricular zone of the cerebral wall. Scale bars: **(A**,**B**,**E**–**I**) 30 μm; (**C**,**D**) 10 μm.

In the cerebral cortex, focal γH2AX-IR was seen in mice older than P15 (Figure 2B–G). The strongest immunocytochemical signal was detected in layer II of the piriform cortex (Figures 2B,D,E and 3I), where labeled nuclei showed high numbers of well defined foci (Figure 2B lower insert). IR was evident also in somatosensory (Figure 2C) and auditory (Figure 2F) cortex. γH2AX-IR was mainly seen in neurons, but also in astrocytes (Figure 2E–G).

In the hippocampus, staining was observed in the subgranular zone of the dentate gyrus at P10–60. In the subventricular zone (SVZ), rostral migratory stream (RMS) and olfactory bulb (OB), γH2AX-positive nuclei were interkinetic, mitotic or, far less frequently, apoptotic. In the wall of the third ventricle, strongest labeling was detected in E 14.5 embryos (Figure 3A); immunoreactivity then declined at birth, but increased again to reach a peak at P10–15. From P20–60, a reduction in the number of γH2AX-positive cells was clearly seen also in individual preparations (Figure 3B). In adult and senescent mice, the number of positive nuclei decreased further (see also Table 1), but IR cells were still scattered throughout the SVZ/RMS/OB (Figure 3C–I).

**Figure 4.**
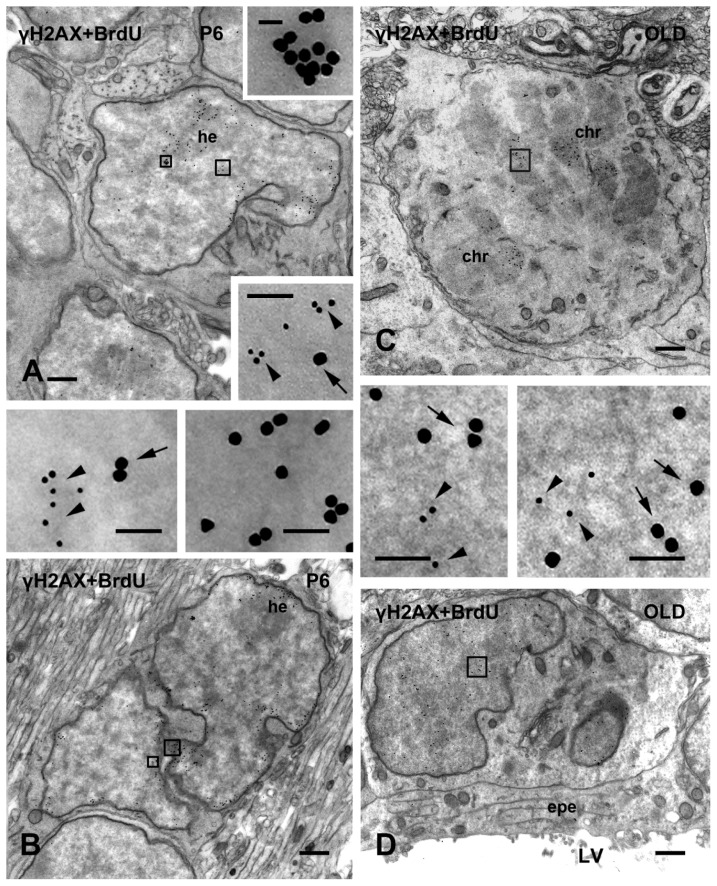
Double immunogold labeling for γH2AX (10 nm gold) and BrdU (20 nm gold) in SVZ. (**A**) Top, double-labeled type C cell with a characteristic deeply indented nucleus and chromatin lax. The areas indicated by the rectangles are shown at higher magnification in the inserts. Bottom, type A cell singularly labeled for BrdU; (**B**) A BrdU singularly-labeled type C cell (top right: right insert at top) and a double labeled type B cell (center: left insert at top); (**C**): Double labeled mitotic cell. The area indicated by the rectangle is displayed in the bottom left insert; and (**D**) Double labeled type C cell. The area in the rectangle is shown at higher magnification in the top right insert. Arrowheads indicate 10 nm gold, arrows 20 nm gold. Abbreviations: γH2AX = phosphorylated form of histone H2AX; BrdU = 5-bromo-2-deoxyuridine; chr = chromosomes; epe = ependyma; he = heterochromatin; LV = lateral ventricle; P = postnatal day. Scale bars: 2 μm; inserts: 100 nm, except top insert in (**A**) 50 nm.

At ultrastructural level (Figure 4), most γH2AX-IR cells in SVZ could be identified as type B (astrocytes) or type C cells (transit amplifying cells).

The relation between H2AX phosphorylation and DNA synthesis was studied after a single injection of BrdU and two-hour survival. In these experiments, most, albeit not all, γH2AX-positive cells in SVZ were also labeled with BrdU, and colocalization between the two labels was confirmed after immunogold labeling (Figure 4). Double IR type A (migrating neuroblasts) and type C cells were seen, but the P5-10 SVZ also contained type B and C BrdU singularly-labeled cells.

To specifically analyze the relationship between γH2AX expression and proliferation, double labeling IMF experiments were carried out with two different antibodies against the phosphorylated form of histone H3 (pHH3) that stains cells in the G_2_ and M phase. In these experiments, all pHH3 positive nuclei in the lateral ventricular wall (Figure 3A,B) were also stained for γH2AX. Most double-labeled nuclei were interkinetic with focal γH2AX nuclear distribution (G_2_ phase). However, M phase cells were also dually stained. As expected, the γH2AX + pHH3 double-labeled nuclei with characteristic M phase morphologies that were detected at E 14.5 belonged to both proliferative populations recognizable in the forming cerebral wall (Figure 3A). γH2AX-IR was demonstrated in proliferating (Figure 3C, yellow; Figure 4C, direct ultrastructural observation) and apoptotic cells (Figure 3D, red) of old mice after IMF or immunogold labeling.

Semi-quantitative data on the colocalization of γH2AX + pHH3 are summarized in Table 2.

An additional quantitative study was carried out in cerebral cortex and SVZ/RMS/OB to compare the relationship of H2AX phosphorylation and DNA synthesis in proliferating (SVZ/RMS/OB) and postmitotic (cerebral cortex) cells. After single or multiple BrdU injection and statistical analysis, the ratio between the numbers of γH2AX + BrdU double IR cells and BrdU-only IR cells was not statistically different among reference sections (see Experimental Section and Figure 6). It was thus possible to simply sum the data obtained from each section and calculate a mean percentage of colocalization for individual animals. Data were then separately analyzed for cerebral cortex and SVZ/RMS/OB. In cerebral cortex, the mean value of γH2AX + BrdU colocalization was 59% (±2.44, *n* = 4) after single and 15% (±7.0, *n* = 4) after multiple BrdU administrations. The difference was statistically significant (Student’s *t*-test, *p* = 0.002). In SVZ/RMS/OB, after a single BrdU injection the mean value of γH2AX + BrdU colocalization was 47.6% (±0.81, *n* = 4). After multiple injections and statistical analysis, a difference was observed between the SVZ/RMS (reference sections A–D) and the OB (reference sections E–H). Data from sections cut through the SVZ/RMS and the OB were summed separately to calculate the percentage of colocalization of individual animals for any of the two areas. The mean value of γH2AX + BrdU colocalization was 24.0% (±3.55, *n* = 4) in the SVZ/RMS, and 10.0% in the OB (±3.55, *n* = 4). These two values were significantly different (Student’s *t*-test, *p* = 0.0098).

#### 2.1.2. Cerebellum

**Figure 5.**
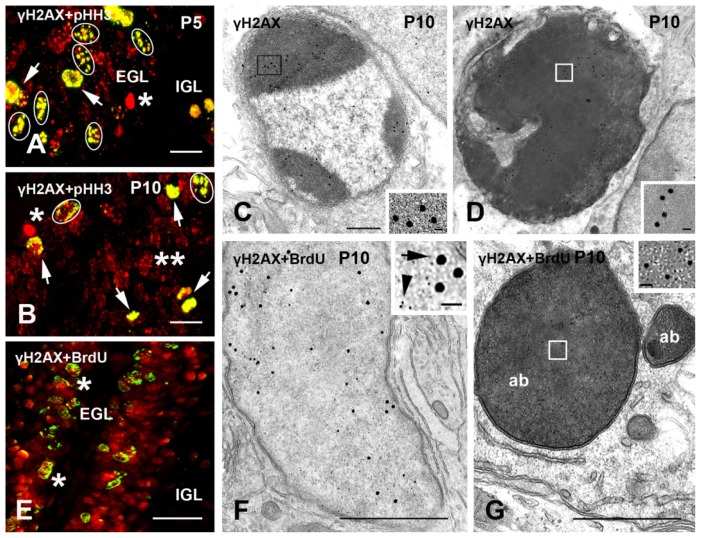
γH2AX-IR in the postnatal mouse cerebellum and its relation with proliferation/DNA synthesis and apoptosis. (**A**,**B**) γH2AX (red) + pHH3 (green) IMF. Double-labeled nuclei (yellow) display M (arrows) and G_2_ phase (ovals) morphologies. Singularly γH2AX-labeled apoptotic nuclei are indicated by an asterisk. The two asterisks in B indicate a γH2AX-IR interkinetic nucleus; (**C**,**D**) γH2AXimmunogold labeling of CGCs in EGL at early (**C**) and mid-to-late stage (**D**) of apoptosis. The areas indicated by the rectangles are shown in the inserts; (**E**) γH2AX (red) + BrdU (green) double staining. Two double-labeled CGCs are indicated by asterisks, but most cells are singularly labeled; and (**F**,**G**) Double immunogold labeling for γH2AX (10 nm gold: arrowhead in insert) and BrdU (20 nm gold: arrow in insert). The CGC in F has a double-labeled interphasic nucleus. Abbreviations: γH2AX = phosphorylated form of histone H2AX; ab = apoptotic bodies; BrdU = 5-bromo-2-deoxyuridine; EGL = external granular layer; IGL = internal granular layer; and P = postnatal day. Scale bars: (**A**,**B**) 10 μm; (**C**,**D**,**F**,**G**) 500 nm; and (**E**) 30 μm; inserts: (**C**,**D**,**G**) 15 nm; and (**F**) 50 nm.

The highest intensity of γH2AX-IR was observed in the external granular layer (EGL) of the postnatal cerebellar cortex (Figure 5).

IR cells reached a peak at P5–10. They declined starting from P15 following the progressive reduction of the EGL. A few γH2AX-positive cells were also seen in the internal granular layer (IGL) (Figure 5A). Ultrastructural examination showed labeling in interphasic (Figure 5F) mitotic, and apoptotic (Figure 5C,D,G) cerebellar granule cells (CGCs). In mitotic CGCs, gold particles were specifically associated with chromosomes, in accordance with double IMF studies. γH2AX-IR apoptotic GCCs displayed specific staining of their nuclei and were at different stages of the apoptotic process (Figure 5C,D,G).

Similar to the SVZ/OB/RMS, the cerebellar cortex displayed colocalization of γH2AX + pHH3 in virtually all proliferating cells, as shown by the extremely low percentages of pHH3 singularly labeled cells in colocalization studies (Table 2). On the opposite, double labeling γH2AX + BrdU IMF experiments showed a limited degree of co-localization (Figure 5E). LM observations were confirmed after immunogold labeling, where γH2AX and BrdU co-localized only in interkinetic CGCs (Figure 5F), while γH2AX-IR mitotic and apoptotic (Figure 5C,D,G) CGCs did not incorporate BrdU.

### 2.2. γH2AX in Proliferating/Apoptotic Cells

This is the first demonstration that H2AX is phosphorylated during embryonic, postnatal and adult neurogenesis in all the areas of the intact mouse brain that display and/or retain proliferative capacity, primarily the SVZ/RMS/OB system and the cerebellar cortex. Notably, quantitative studies in these areas demonstrated a very high degree of colocalization (up to 90%) between γH2AX and the cell proliferation marker pHH3 in IMF, and gold particles indicative of γH2AX-IR were directly observed in M phase neurons at the transmission electron microscope (TEM). At both light and ultrastructural levels, γH2AX-IR in interkinetic nuclei was mainly observed to occur in foci, albeit diffuse staining of the nucleoplasm was also evident, particularly at TEM. These observations were in part surprising since focal phosphorylation of H2AX is currently believed to be primarily related to DNA damage [1,2,4,16,17,24], and a global pan-nuclear γH2AX DNA staining has previously been observed in M phase cells [12].

γH2AX was detectable from embryonic life to senescence in the SVZ/RMS/OB system. It is a well-established notion that in the (mouse) brain, the SVZ is a neurogenic stem cell niche that persists throughout life, albeit progressively reducing with senescence [25–27]. Numerous studies on the SVZ/RMS/OB system have used (and often misused) the S phase marker BrdU to detect proliferating cells. The striking difference in the degree of colocalization of γH2AX with the “true” proliferation marker pHH3 from one side and BrdU from the other indicates that a substantial number of S phase cells in the SVZ/RMS/OB system express γH2AX, suggesting that BrdU incorporation may, in these cells, be related to a DDR. A reduction of BrdU incorporation in the SVZ of elderly mice was previously reported, and it was speculated that an increased cell death was responsible for lowering the number of S phase cells in the elderly [27]. Apoptotic cells in the SVZ/RMS/OB were so rare in our material, and we found no direct evidence for an increase of apoptotic cells in the senescent mouse SVZ. However, after multiple injections of BrdU we demonstrated a statistically significant difference in the percentage γH2AX + BrdU double-labeled cells in the SVZ/RMS (the proliferative compartment) compared to the OB (the receptive compartment), with a reduction to 0.41 fold in the latter. Migration of neural precursors from SVZ to the OB along the RMS is a well-established phenomenon [25–27]. Therefore, the relative disappearance of γH2AX in the foremost part of the system indicates that effective DDR mechanisms occur as migrating cells travel towards the OB.

Another site of the brain where neurogenesis is paralleled by an intense phosphorylation of H2AX is the developing cerebellar cortex, wherein actively mitotic precursors generate the CGCs that migrate eventually to the IGL. Here, an important neurogenetic wave occurs during the first two postnatal weeks, in parallel with the peak of H2AX phosphorylation. Therefore, the time course of γH2AX-IR displays a direct correlation with the process of CGC differentiation/maturation [28], so that the degree of colocalization between γH2AX-IR and pHH3 reaches values of 80%–90%. Differently from the SVZ/RMS/OB system, γH2AX-IR could also be easily detected in CGCs with the typical ultrastructural features of early and late apoptosis. In keeping with our previous studies demonstrating that apoptotic cell death rapidly follows proliferation in a large population of CGCs [28], γH2AX immunoreactivity was observed in these neurons together with the S phase marker BrdU as soon as after two hours from administration of the tracer (Figure 5G). Thus our data indicate γH2AX as an important signal for the cell to commit itself to apoptosis, and lead to speculate that H2AX phosphorylation, besides being one of the first DDR signals, may subsequently represent a marker of the persistence of residual DNA damages. This study unequivocally demonstrates that H2AX is phosphorylated in proliferating neurons. However, it leaves open the question whether or not activation of H2AX is indeed related to the occurrence of DNA DSBs during cell division, apoptosis or, simply, to physiological activity, as recently demonstrated in hippocampus [29]. Hematopoietic stem cells or jejunum epithelial stem cells display strong levels of DSB repair activity under normal conditions [30,31]. Therefore, it is possible that similar events also occur in proliferating neurons. The possibility that neuronal activation of H2AX simply occurs during mitosis (*i.e.*, strictly speaking, the M phase) [12] can, instead, be ruled out on the basis of our observations, although H2AX has also been implicated, together with HH3, in chromosome condensation [32]. Although the biological function of H2AX during mitosis (if any) remains to be elucidated in full, it was recently shown that its phosphorylation blocks DNA synthesis and cell cycle progression in neural stem cells, and that their self-renewal and the niche size can be dynamically modulated by targeting H2AX activation [33,34].

Given that a block of cell cycle progression is often associated with cell death in neurons, the occurrence of γH2AX in apoptosis [1,35] deserves further discussion. Initial studies demonstrated that apoptotic DNA fragmentation resulted in H2AX phosphorylation as a DDR [36], suggesting that H2AX activation is a consequence of apoptosis. However, other experiments indicated that, instead, activation of H2AX may precede apoptosis rather than follow it, because H2AX contributes to the apoptotic process by altering the chromatin conformation and increasing the accessibility of DNA to apoptotic effectors [36,37]. In the case of the CGCs, where close temporal links (as short as twenty four hours) have been demonstrated between DNA synthesis (S phase) and apoptosis [28], localization of γH2AX in early apoptotic cerebellar neurons suggests that phosphorylation of H2AX occurs once CGCs are already committed to death. This is further reinforced by the colocalization of γH2AX + BrdU in CGCs with a normal interkinetic nucleus, as we have previously demonstrated that some of these neurons display a fragmented DNA in the presence of an ultrastructurally unaltered nucleus [28]. It is worth mentioning here that a link between apoptosis and a deregulated cell cycle has been implicated in the pathophysiology of different neurodegenerative diseases [38]. Therefore, it is tempting to speculate that targeting H2AX might represent a promising neuroprotective strategy to pursue for the future.

### 2.3. γH2AX in Postmitotic Cells

The cerebral cortex resulted in being a major site of expression of γH2AX in fully mature and senescent mice. For the most, γH2AX immunoreactivity was detected in nuclei belonging to NeuN-positive neurons, but also some GFAP-IR astrocytes had a positive nucleus. In both types of cells, IR nuclei were interkinetic with a focal distribution of γH2AX. There was no co-localization with the proliferation marker pHH3. The only occasional pHH3 singularly labeled nuclei belonged to non-neural cells, *i.e.*, some endothelial cells in blood vessels.

In the cerebral cortex of senescent animals, the exclusively focal appearance of γH2AX-IR and the high percentage of γH2AX + BrdU colocalization (about 60%) indicate that BrdU incorporation is primarily related to a DDR occurring in postmitotic neurons and glial cells. Quantitative analysis demonstrated that the percentage of cortical cells expressing the two labels *versus* the total number of BrdU-labeled cells underwent minimal fluctuations along the rostro-caudal axis of telencephalon. This indicates that the senescent cerebral cortex, as a whole, is prone to DNA damage, very likely in the form of DSBs. Further experiments are needed to definitively prove or disprove the occurrence of DSBs, as it was previously shown—albeit in a transformed fibroblast cell line—that cellular senescence can also be associated with a *pseudo* DDR, *i.e.*, γH2AX foci in the absence of detectable DNA breaks [39].

Data on the temporal distribution of H2AX phosphorylation after ionizing radiations demonstrated that, after a short plateau of approximately one hour, the number of γH2AX foci started to decrease two hours post-irradiation and returned to baseline within six hours [40]. Although these data are difficult to compare with the situation *in vivo*, they are in line with the notion that γH2AX rapidly returns to baseline once repair has been successful. Therefore, we can reasonably assume that cortical cells, which incorporated BrdU but were negative to γH2AX, are committed to death, in agreement with the known intervention of γH2AX in the modulation of checkpoint responses [41]. DNA repair systems in the absence of an external insult should be of great importance in the adult and old brain [42–44], and efficient DNA repair is needed in long-living cells with no or limited regeneration from precursors, such is the case of adult/senescent cortical neurons (and glia). Molecular/pharmacological targeting of these systems has a good potential for development of novel neuroprotective strategies. In cells with a very long life span such as neural cells, adipocytes and muscle fibers, a common paradigm of DSB repair has been observed after biochemical and *in vitro* experiments. Similarly to neurons, adipocytes have a very slow turnover in the adult [45] and a study on the pre-adipocyte cell line 3T3F442A demonstrated that DNA repair activity increased in postmitotic cells, in opposition to the view that DSB repair was decreased during differentiation [46]. An active process of protection against DNA DSBs was also observed in differentiated myotubes. It counteracted the consequences of DNA damage, but was not detected in myoblasts, the less differentiated precursors of the muscle fibers [47]. Thus, it was hypothesized that differentiated long-lived cells such as the neurons and the muscle fibers acquire active mechanisms that hamper the effects of DNA DSBs as compared to their less-differentiated precursors [47,48]. The work presented here gives strong support to this hypothesis.

## 3. Experimental Section

### 3.1. Animals

All experimental procedures were approved by the Italian Ministry of Health and the Committee of Bioethics and Animal Welfare of the University of Torino, Torino, Italy.

Labeling of DNA synthesis *in vivo* was carried out as follows. BrdU (Sigma, St. Louis, MO, USA) was dissolved in sterile water at 100 mg/mL and injected intraperitoneally at 0.1 mg/g body weight. A single BrdU injection was given to animals [two postnatal day 6 (P6), two P10 and two 24 months old mice for electron microscopy (EM) studies, and six 24 months old mice for light microscopy (LM) studies] followed by a two-hour survival. Six additional 24 months old mice received a daily BrdU injection for one week, followed by sacrifice twenty four hours after the last administration of the tracer, and were employed for LM studies. The immunocytochemical visualization of γH2AX-IR was performed on embryonic (E 14.5), postnatal (P0, P5, P10, P15, and P20), adult (P60), and senescent (18, 24 months) mice (four at each age). Three mice for each postnatal age (P0, P5, P10, P15, P20, and P60) were used for detection of γH2AX in brain tissue homogenates.

### 3.2. Immunocytochemistry

Animals (untreated or treated with BrdU) were deeply anesthetized with an intraperitoneal injection of sodium pentobarbital (30 mg/kg), and perfused with 4% paraformaldehyde (PFA) in 0.1 M phosphate buffer (PB) for LM or 2% glutaraldehyde + 1% PFA in 0.05 M sodium cacodylate buffer for EM. After dissection, brains were left in fixative for two hours. Embedding (paraffin or Araldite) and cutting were carried out according to standard procedures.

The light microscopy ICC detection of γH2AX and related molecules was performed by the ABC method or double IMF.

The semi-quantitative analysis of the distribution of γH2AX reported in Table 1 was carried out by counting, for each animal, the number of γH2AX-IR nuclei in individual areas of the brain from five coronal sections cut through the forebrain (P60/OLD: bregma −2.18, −1.22, 0.98, 1.34, and 3.56; see also Figure 1), one section cut through the midbrain (P60/OLD: bregma between −4.04 and −4.60), and one section cut through the hindbrain (P60/OLD: bregma between −6.00 and −6.64). Parasagittal sections of the brain *in toto* were used in E 14.5 embryos. Results were averaged for each group of age, and reported according to the following scale: − = no signal, *i.e.*, 0 IR nuclei; + = weak staining, *i.e.*, ≤10 IR nuclei; ++ = moderate staining, *i.e.*, >10 and ≤50 IR nuclei; +++ = strong staining, *i.e.*, >50 IR nuclei.

The percentages of γH2AX singularly-labeled cells, pHH3 singularly-labeled cells, and γH2AX + pHH3 double-labeled cells were calculated at different ages after double IMF on randomly selected sections from at least three different animals/age. At least 250 immunolabeled cells per age were directly observed under the confocal microscope.

Electron microscopic ICC was performed using single and double immunogold standard labeling procedures.

### 3.3. Western Blotting

Brains were quickly removed and frozen in liquid nitrogen, powdered and suspended in 2.5 volumes of a cold general lysis buffer (50 mM Pipes, 300 mM Sucrose, 100 mM NaCl, 5 mM EGTA, 5 mM HgCl_2_, 100 μM ZnCl_2_, 1mM Sodium orthovanadate, Triton X-100 1%, pH 6.8) containing protease inhibitors (1 mM PMSF, and 5 mg/mL aprotinin). The homogenate was sonicated and centrifuged at 1.2 × 10^4^ *g*/min at 4 °C for 10 min. The immunological detection of γH2AX in brain extracts was carried out after immunoprecipitation following standard procedures.

### 3.4. Primary Antibodies

Table 3 reports a list of primary antibodies, with characteristics, conditions of use and relevant references where available.

### 3.5. Quantitative Studies on γH2AX + BrdU Colocalization in Senescent Mice and Statistics

**Figure 6.**
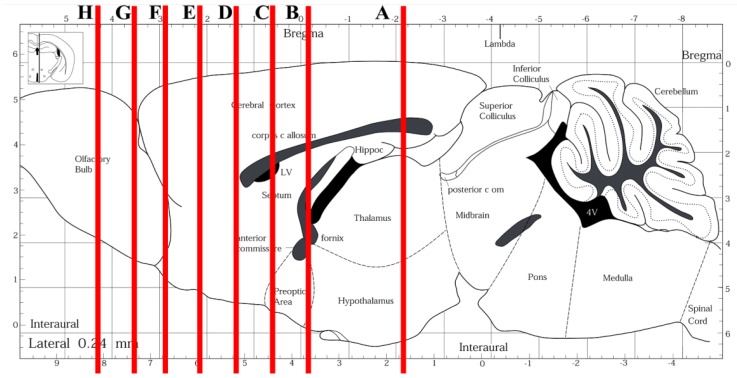
Coronal reference sections used for quantitative studies of γH2AX + BrdU colocalization in senescent mice. Red bars (**A** to **H**) indicate the stereotaxic coordinates of individual coronal sections with respect to a sagittal section of the adult mouse brain (adapted from [23]). For anatomical mapping, the entire forebrain was serially cut along the rostro-caudal axis at 6 μm, and coronal sections at 750 μm intervals were stained with toluidine blue for identification of anatomical reference structures. The eight reference points were identified as follows: **A**, hippocampus (bregma −2.18 mm); **B**, interventricular foramem (bregma −0,22 mm); **C**, lateral ventricle at the point of alignment with the anterior arm of corpus callosum (bregma 0.62 mm); **D**, maximal reduction of lateral ventricle extension, corresponding to start of the RMS (bregma 1.34 mm); **E**, RMS at the level of intersection of rhinal fissure with a line tangent to the lateral margin of the intrabulbar portion of the anterior arm of corpus callosum (bregma 2.10 mm); **F**, end of RMS at the level of first stratification of olfactory bulb (bregma 2.80 mm); **G**, olfactory bulb at the level of the rostral margin of the accessory olfactory bulb (bregma: 3.56 mm); and **H**, olfactory bulb (bregma 4.28 mm).

Quantitative studies on γH2AX + BrdU colocalization were carried out in series of eight sections cut through the forebrain of senescent mice (Figure 6). Eight slides/animal (each bearing the three serial sections that immediately followed the toluidine blue-stained reference sections) were used for double immunocytochemical labeling quantitative studies. Single and double-labeled immunoreactive cells were directly counted in a single plane of focus with a DM6000B fluorescence microscope (Leica Microsystems, Weitzlar, Germany) at 40× magnification.

Statistics was carried out with the SPSS software (IBM, Armonk, NY, USA). For γH2AX + BrdU colocalization, mean percentages of labeled cells in experimental animals were compared with one-way ANOVA and the Student’s *t*-test with the Bonferroni correction. Differences were considered significant for *p* < 0.05.

## 4. Conclusions

This study demonstrates that γH2AX is expressed in the mouse brain from embryonic life to senescence in the absence of experimentally evoked damage to cellular DNA. Focal and non-focal phosphorylation of H2AX in neurogenetic areas of forebrain and cerebellum is linked to cell proliferation, which, in neural stem cells, has been previously shown to be regulated in a different manner from most somatic cells with a fundamentally distinct mechanism of DDR and checkpoints [33]. On the other hand, in cerebral cortex of senescent mice the appearance of γH2AX-IR foci is most likely related to DSB occurrence and repair. As excessive glutamate stimulation [19,20] increased activity, and β-amyloid [29] can induce DSBs, our study indicates that H2AX phosphorylation may be a response of the normal postmitotic neurons to an array of physiological and/or pathological situations eventually leading to a DDR and/or a pseudo DDR.

Therefore, targeting H2AX and/or DDR-related molecules looks to be a promising strategy to develop new approaches to counteract neurodegeneration, as the loss of function of DDR proteins (among which γH2AX) appears to be tightly linked to several neurodegenerative diseases [55].

## Figures and Tables

**Table 1.** Semi-quantitative distribution of γH2AX-IR nuclei in the mouse brain. Staining was performed on paraffin sections using the ABC method. The level of immunoreactivity (IR) through the brain was assessed using the following scale: − = no signal; + = weak staining; ++ = moderate staining; +++ = strong staining. Abbreviations: γH2AX = phosphorylated form of histone H2AX; E = embryonic day; OB = olfactory bulb; P = postnatal day; RMS = rostral migratory stream; and SVZ = subventricular zone.

Distribution of γH2AX-IR nuclei in mouse brain								
Age	E 14.5	P0	P5	P10	P15	P20	P60	Old
Forebrain								
Cerebral cortex	−	−	−	−	+	++	+++	+++
SVZ/RMS/OB	+++	+	+	+++	++	++	++	++
Amygdala	−	−	−	−	+	+	++	−
Hypothalamus	−	−	−	−	−	+	++	−
Dorsal endopiriform nucleus	−	−	−	+	−	+	+	−
Hippocampus	−	−	−	+	+	+	+	−
Midbrain	−	−	−	−	+	+	+	−
Hindbrain								
Cerebellum	−	+	+++	+++	+	−	−	−
Pons	−	−	−	−	+	+	+	+

**Table 2.** Percentages of γH2AX singularly-labeled nuclei, pHH3 singularly-labeled nuclei and γH2AX + pHH3 double-labeled nuclei in the SVZ in SVZ/RMS/OB and cerebellum after double IMF. At least 250 IR nuclei per age were directly observed under the confocal microscope and ascribed to any of the three categories. Abbreviations: γH2AX = phosphorylated form of histone H2AX; E = embryonic day; P = postnatal day; and pHH3 = phosphorylated form of histone H3.

Quantification of γH2AX + pHH3 IR nuclei in mouse SVZ/RMS/OB and cerebellum						

Age	% of IR nuclei					

	SVZ/MS/OB			Cerebellum		

	γH2AX only	pHH3 only	Colocalization	γH2AX only	pHH3 only	Colocalization
E 14.5	29.4	0	70.6			
P0	22.8	0.8	76.4	18.8	1	80.2
P5	33.8	0	66.2	21.1	0.3	78.6
P10	22.2	1.2	76.6	8.4	1.6	90.0
P15	25.5	0	74.5			
P20	26.6	0	73.4			
P60	27.3	0	72.7			

**Table 3.** Primary antibodies. Abbreviations: γH2AX = phosphorylated form of histone H2AX; BrdU = 5-bromo-2-deoxyuridine; GFAP = glial fibrillary acidic protein; ICC EM = immunocytochemistry electron microscopy; ICC LM = immunocytochemistry light microscopy; M = mouse; NA = not available; NeuN = Neuronal nuclei antigen; pHH3 = phosphorylated form of histone H3; R = rabbit; and WB = Western blotting.

Primary antibodies						
Antibodies	Target/epitope	Type	Dilution	Use	Supplier	Refs
γH2AX (Ser139)	aa 134–142 of human H2AX (C-KATQA[pS]QEY)	Mono, M	1:200	ICC LM	Upstate Biotechnology, Lake Placid, NY, USA	NA
γH2AX (Ser139)	aa 134–142 of human H2AX (C-KATQA[pS]QEY)	Poly, R	1:500	ICC LM WB	Millipore, Billerica, MA, USA	NA
γH2AX (Ser139)	Residues surrounding Ser139 of human H2AX	Poly, R	1:25	ICC EM	Cell Signaling Technology, Danvers, MA, USA	NA
pHH3	aa 1–100 of human HH3, phosphorylated at Ser10	Poly, R	1:2000	ICC LM	Abcam, Cambridge, UK	[49]
pHH3	aa 7–20 of human HH3 (ARKpSTGGKAPRKQLC)	Poly, R	1:1000	ICC LM	Upstate Biotechnology, Lake Placid, NY, USA	[50]
BrdU	Exogenously administered BrdU	Mono, M	1:5	ICC LM	GE Healthcare, Chalfont St. Giles, UK	[51]
BrdU	Exogenously administered BrdU	Mono, Rat	1:10	ICC EM	AbD Serotec, Oxford, UK	[52]
NeuN	Purified neuronal nuclei from mouse brain	Mono, M	1:100	ICC LM	Millipore (Chemicon), Billerica, MA, USA	[53]
GFAP	GFAP purified from bovine spinal cord after Triton X-100 extraction	Poly, R	1:2000	ICC LM	Abcam, Cambridge, UK	[54]
